# Traumatic Corneal Abrasion

**DOI:** 10.7759/cureus.4396

**Published:** 2019-04-05

**Authors:** Nicholas Fusco, Tej G Stead, David Lebowitz, Latha Ganti

**Affiliations:** 1 Emergency Medicine, Osceola Regional Medical Center, Kissimmee, USA; 2 Emergency Medicine, Brown University, Providence, Rhode Island, USA; 3 Emergency Medicine, University of Central Florida College of Medicine, Orlando, USA; 4 Emergency Medicine, University of Central Florida College of Medicine / Hospital Corporation of America Graduate Medical Education (HCA GME) Consortium, Kissimmee, USA

**Keywords:** corneal abrasions, eye trauma

## Abstract

Corneal abrasions can have potentially sight-threatening consequences if not accurately diagnosed and managed appropriately in the acute period. Simple corneal abrasions can be managed with antibiotic and tetanus prophylaxis, analgesia, and next-day follow up with ophthalmology. However, if there is any suspicion for penetrating eye injury, corneal ulcer, a sight-threatening infection such as bacterial keratitis, or ophthalmic zoster, an emergent referral is imperative. In this report, we present a case of classic corneal abrasion and discuss the acute management of this common problem.

## Introduction

Eye-related problems are a common presentation in the emergency department (ED), accounting for approximately 8% of cases [1], with corneal abrasions representing almost half of the diagnoses [2]. As such, being able to diagnose and manage a corneal abrasion acutely and knowing when to refer is imperative. We present a classic case of traumatic corneal abrasion and discuss its ED management and associated controversies.

## Case presentation

A 29-year old male presented to the ED with chief complaint of pain in the right eye, inability to open the eye, and excessive tearing in the right eye. He stated that, one day before arriving at the ED, his 3-year-old child poked him with a wooden skewer in his right eye. There was no active bleeding in the eye. He denied experiencing fever, chills, headache, nausea, or vomiting. Remainder of the review of systems was negative. The patient did not use glasses or contact lenses. His past medical and surgical history were noncontributory.

**Figure 1:**
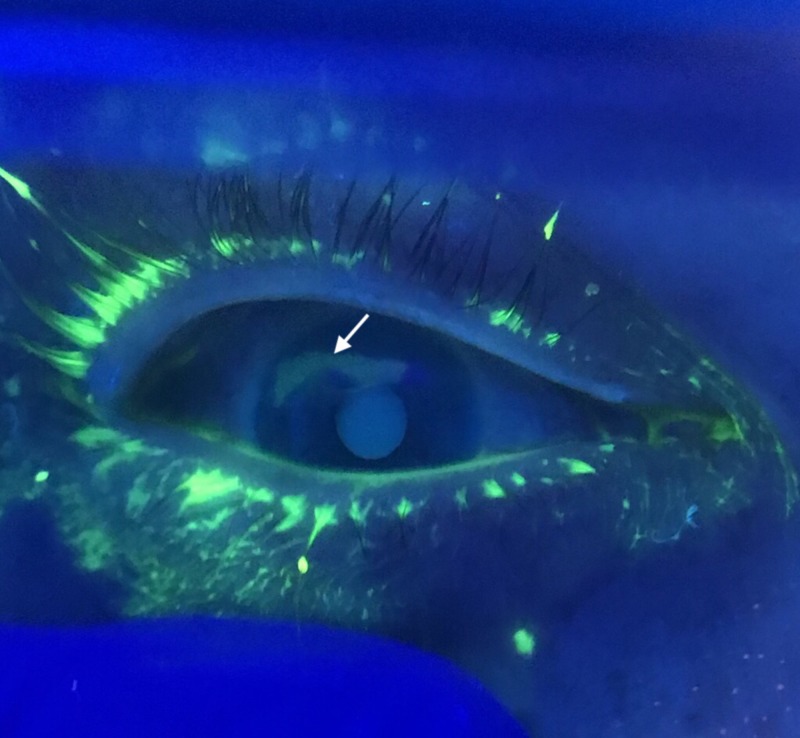
Fluorescein staining of the eye revealing a large corneal abrasion at the superior aspect (arrow)

His vital signs were pulse 72, blood pressure 142/78 mmHg, temperature 37.2° C, respirations 17/min, and pulse oximetry 99%. Visual acuity was 20/50 in his left eye and 20/70 in his right eye. Intraocular pressure (IOP) in the left eye was 18 mmHg and 20 mmHg in the right eye (normal IOP is less than 22 mmHg). Pupillary shape was normal. There was no hyphema, and Seidel’s test was negative. Fluorescein staining revealed an abrasion at the superior aspect of the cornea, as depicted in Figure 1. The patient was discharged home with pain control and erythromycin antibiotic ointment. He was instructed to follow up with his primary care physician and ophthalmology within 24 hours, and to return to the ED should symptoms worsen.

## Discussion

Etiology

Corneal abrasions are most commonly caused by direct minor trauma, accounting for 64% of cases, and as was the case with our patient; 12% of these are caused by contact lens wear, thus accounting for 19% of those cases caused by minor trauma [3]. Corneal abrasion can be identified by the following symptoms: eye pain, tearing, decreased visual acuity, photophobia, red eye, and the sensation of a foreign body [1]. Risk factors include a history of trauma, contact lens use, male sex, age between 20 and 34 years old, a construction or manufacturing job, and lack of eye protection [3].

Evaluation

As with any eye complaint, a detailed but focused history and physical are obtained. Eye examination includes visual acuity and pressure measurements, followed by fluorescein testing. Fluorescein is a dye that stains any defects in the cornea orange, and the clumping is magnificently evident in cobalt blue light or with the light of a wood’s lamp. A positive Seidel’s test is “leaking” of the fluorescein dye from the anterior chamber, which can be seen with full or partial thickness corneal tears with or without globe rupture. A positive Seidel’s test is an ophthalmologic emergency [4].

Treatment

To prevent infection, antibiotic (usually erythromycin) ointment is prescribed for non-contact lens wearers. The ointment has the additional benefit of “coating” the eye with a thin protective film. The flip side is the blurry vision that the patient may find bothersome. For contact lens users, additional antibiotic prophylaxis with ciprofloxacin or ofloxacin or tobramycin drops is given to cover pseudomonas [1]. If contaminated objects such as vegetative or fecal matter cause the corneal abrasion, bacterial keratitis can occur which is an infection that requires ophthalmologic management.

In addition to antibiotics for infection, tetanus prophylaxis should be given, as the corneal abrasion is a tear in the cornea and is thus a portal of infection. For analgesia, a cycloplegic agent can be given to prevent the eye from dilating and constricting in response to light, which can be painful. More recently, ketorolac for the eye had become available and these topical non-steroidal anti-inflammatory drug (NSAID) drops are effective in alleviating eye pain without causing gastrointestinal upset. There is no role for opiates in the management of pain associated with corneal abrasions.

Topical anesthesia with tetracaine drops is frequently provided in the ED; however, the traditional teaching is that patients cannot be sent home with these very effective pain relievers, due to the fear that they result in poor wound healing of the corneal epithelium. This myth was recently debunked [5] when the evidence for this claim was examined. The evidence consisted of individual case reports and small case series, and in most cases, the patients had been using the topical anesthetics for weeks to a month in higher doses than used in the ED. Four clinical trials have subsequently evaluated the safety and efficacy of topical anesthetics versus placebo, and found that topical anesthetics did a better job of controlling pain and showed no statistical difference in cornea epithelial healing at 72 hours. Thus, prescribing a few days of topical anesthetic is considered safe.

Patching of the eye is sometimes recommended as a way to prevent photophobia and the resulting pain from ciliary accommodation. In a systematic review of 12 trials that randomized a total of 1080 participants in five countries (United Kingdom, United States of America, Canada, Brazil, and Switzerland), no clear evidence of benefit (or harm) with eye patching was found [6]. 

Referral

Patients with simple corneal abrasions can be managed in the ED initially with referral to ophthalmology within 24-48 hours. Any evidence of a corneal tear, threat to vision, or abnormal intraocular pressures warrant immediate ophthalmologic consultation.

## Conclusions

In this case, the authors highlight the management of an acute traumatic corneal abrasion. The mainstay of treatment includes analgesia and prophylaxis against infection. Patching is not currently recommended, and the fear of prescribing short-term topical anesthetics (until ophthalmology follow up within 72 hours) is unwarranted. 
